# United Forces of Botanical Oils: Efficacy of Neem and Karanja Oil against Colorado Potato Beetle under Laboratory Conditions

**DOI:** 10.3390/plants8120608

**Published:** 2019-12-14

**Authors:** Kateřina Kovaříková, Roman Pavela

**Affiliations:** Group Secondary Metabolites in Crop Protection, Crop Research Institute, Dmovská 507, 161 06 Praha 6-Ruzyně, Czech Republic; pavela@vurv.cz

**Keywords:** synergism, neem, karanja, Colorado potato beetle, botanical insecticides

## Abstract

Neem and karanja oil are the most promising botanical insecticides in crop protection nowadays. Given that information about the insecticidal abilities of these oils is lacking, the aim was to explore the effects of neem and karanja oil binary mixtures. The insecticidal activity of NeemAzal T/S (Trifolio-M GmbH, Lahnau, Germany) (neem oil), Rock Effect (Agro CS a.s., Česká Skalice, Czech Republic) (karanja oil), and their binary mixes (at 1:1, 1:2, and 2:1 volume ratios) against the larvae of the Colorado potato beetle (CPB; *Leptinotarsa decemlineata*) was studied. In our bioassays, a synergistic effect of the mixtures, which was dose-dependent, was observed for the first time against this pest. The most effective blend was the 1:1 ratio. Its efficacy was more or less the same as, or even greater than, the neem oil alone. The LC_50_ of neem oil two days after application was (0.075 g·L^−1^) and the LC_50_ of the mixture was (0.065 g·L^−1^). The LC_50_ of karanja oil was (0.582 g·L^−1^), which was much higher than the LC_50_ of neem oil. The LC_90_ of neem oil five days after application was (0.105 g·L^−1^) and the LC_90_ of the mixture was (0.037 g·L^−1^). The LC_90_ of karanja oil was (1.032 g·L^−1^). The results demonstrate that it is possible to lower the doses of both oils and get improved efficacy against CPB larvae; nevertheless, further verification of the results in field conditions is necessary.

## 1. Introduction

The Colorado potato beetle (CPB), (*Leptinotarsa decemlineata* (Say, 1824), Coleoptera: Chrysomelidae) is one of the most important potato pests. This species is native to North America, from where it has gradually spread across Europe and Asia [1] alongside the expansion of potato cultivation. *L. decemlineata* is now considered to be the most important insect defoliator of potatoes. Through defoliation, the yield of tubers can be reduced by more than 50% [2]. Moreover, if the pest appears early and in intense numbers, it can destroy the entire production of a potato growing operation. The values of “intense numbers” vary from author to author and correspond with economic (action) thresholds, which were established for optimizing the use of insecticide applications and are unique to certain conditions. For example, Senanayake and Holliday [3] suggest 0.14 to 0.82 larvae per plant. Mailloux and Bostanian [4] stated that a measure based on the level of defoliation is better than one based on pest abundance. These levels were estimated, for example, by Zehnder et al. [5] at 20% leaf loss for young plants, 30% for plants from early bloom to late bloom, and 60% for plants from late bloom to harvest for fields in eastern Virginia. Nevertheless, the CPB larval stage is considered to be the most harmful stage to potato production [4], so we focused our research on the CPB larvae. 

Various non-chemical control methods have been introduced since the 19th century to reduce the impact of CPB. Crop rotation, trap crops (eggplant), and other agrotechnical practices were recommended to farmers at that time [6]. In a study by Reed [6], it was concluded that the only two reliable methods were hand picking and the use of Paris Green (a toxic pigment). Hand picking, especially before mating, was considered very effective, but impractical on a larger scale. As such, the majority of alternative methods failed, and the most common method became the use of pesticides. Although pests have developed resistance to many active ingredients of insecticides, the use of chemicals remains the most widely used method against CPB to date (although biological controls are also often applied [7,8,9]).

According to the Arthropod Pesticide Resistance Database [10], CPB has shown resistance to 56 active ingredients of insecticides to date, among them spinosad [11] and *Bacillus thuringiensis* [12]. Mota-Sanchez et al. [11] also observed cross resistance to nine other neonicotinoids in an imidacloprid-resistant adult population of CPB. As of now, resistance has been recorded to most of the commonly used insecticides, such as pyrethroids, neonicotinoids, diamides, and organophosphates. As Yamamoto et al. [13] stated, it is possible to delay or prevent resistance development of pests via rotating insecticides with different modes of action and using certain combinations of insecticides. Barnes et al. [14] proved that a strategy using mixtures of insecticides is even more effective than the rotation of insecticides, and this is the very essence of botanical insecticides (BIs), which are complex mixtures of many functional secondary metabolites [15,16], in contrast to chemical substances, which are often characterized by one active ingredient, complemented by a number of inactive ingredients to facilitate the application. Moreover, the excessive use of chemical pesticides harms the environment, non-target organisms, and humans. By contrast, botanical pesticides are biodegradable and leave no harmful residues. Unfortunately, the scale of products applicable in organic production is insufficient as well. It is necessary to search for additional environmentally acceptable substances for effective control of CPB, and the use of botanical pesticides is a promising possibility. Chaudhary et al. [17] stated that the use of botanical pesticides is the most efficient means to replace synthetic pesticides, and among those, extracts and oils are the best choices. Neem and karanja oil, in particular, have great potential for use in sustainable integrated pest management.

Neem oil is one of the most promising substances in the current approach to pest control. Neem oil is a product of the Indian neem tree *Azadirachta indica* (A.) Juss. It possesses a variety of insecticidal properties, such as repellency [18], antifeedancy, toxicity, and growth disruption, against numerous pest species [19]. For example, the biochemical effect (growth inhibition, feeding deterrence, oviposition inhibition) of neem oil against more than 30 Lepidopteran pests [20] has been well documented. The main active ingredient is azadirachtin, a tetranortriterpenoid, which was isolated from the seeds of *Azadirachta indica* by Butterworth and Morgan [21] and is known to disrupt insects’ metamorphosis [22]. Neem oil is a contact insecticide, but even systemic activity has been documented [23]. Pavela et al. [24] also proved that azadirachtin can be taken up by plant roots and thus affect the population of immature aphids feeding on the treated plant.

Karanja oil is a product of the seeds of a widespread tropical and subtropical tree called *Pongamia pinnata* (L.) Pierre [25]. Karanja oil is rich in furano-flavonoids [26]. Al Muqarrabun et al. [27] summarized the known attributes of up to 70 flavones and their derivatives that had been isolated from *Pongamia pinnata* by various authors. Of these, karanjin, which was first discovered by Limaye [28], is particularly effective against a large number of insects [29]. The oil and extract of karanja act as insecticides [30], repellents, antifeedants, and growth regulators [31], and even oviposition deterrents [32]. 

Some insecticides, in combinations, may exhibit greater-than-additive toxicity, but the prediction of mixture toxicity using a response addition model is not always accurate for active ingredients with different modes of action [33]. In the case of neem and karanja oils, a synergistic effect was found by Kumar et al. [34] in bioassays on aphids and mites. Later, this phenomenon was confirmed by Packiam and Ignacimuthu [35] during experiments on *Spodoptera litura*. However, although the synergistic effect of insecticides is of great importance in practice, there is still much to be explored in this research area; for example, application doses may be reduced (economic and environmental benefits) and, moreover, multi-substance mixtures prevent the development of resistant populations of pests (anti-resistant strategy). 

Although the efficacy of neem extracts on the Colorado potato beetle has already been studied, the efficacy of karanja oil against this pest has not yet been reported. Similarly, a possible synergistic effect of binary mixtures of these oils has also not been studied sufficiently to date. Thus the aim of our research was to evaluate the insecticidal properties of two commercial products (NeemAzal T/S - Trifolio-M GmbH, Lahnau, Germany, Rock Effect - Agro CS a.s., Česká Skalice, Czech Republic)), based on *Azadirachta indica* seed kernel oil and *Pongamia pinnata* oil, respectively, against the most destructive stage of CPB and to verify any possible synergistic effects of their binary mixtures in three different ratios with regard to practical use.

## 2. Results

The insecticidal activity of both tested BIs against CPB larvae was very good; however, significant differences in efficacies were found. From the LC_50_ (LC_90_) of Rock Effect (Agro CS a.s., Česká Skalice, Czech Republic) (0.582 g·L^−1^) and NeemAzal T/S (Trifolio-M GmbH, Lahnau, Germany) (0.075 g·L^−1^), it is obvious that karanja oil by itself is significantly less effective against larvae of *Leptinotarsa decemlineata* in comparison with neem oil. Complete results are presented in Table 1.

A comparison of the LC values revealed that both BIs showed significant chronic toxicity, which resulted in an apparent decrease in lethal concentrations over time (Table 1). The LC_50_ (LC_90_) values for NeemAzal T/S (Trifolio-M GmbH, Lahnau, Germany) were estimated at 0.075 g·L^−1^ (0.618 g·L^−1^) after 48 h and 0.005 g·L^−1^ (0.029 g·L^−1^) after 8 days, which is an obviously lower range of concentrations than the estimate for Rock Effect (Agro CS a.s., Česká Skalice, Czech Republic), where the LC_50_ (LC_90_) was 0.582 g·L^−1^ (1.692 g·L^−1^) after 48 h and 0.259 g·L^−1^ (0.774 g·L^−1^) after 8 days.

The insecticidal activity of the mixtures was assessed by the activity of the pure BIs. The LC range of the mixtures was usually within the LC range of NeemAzal T/S (Trifolio-M GmbH, Lahnau, Germany) and Rock Effect (Agro CS a.s., Česká Skalice, Czech Republic). As such, all the mixtures were more efficient than Rock Effect (Agro CS a.s., Česká Skalice, Czech Republic) alone. An exception was found in the case of mixtures after 48 h (acute toxicity), when the LC_90_ was higher for the mixtures than for the original substances. The LC values of the mixtures decreased over time, as did the LC values of the pure BIs. The LC_50_ (LC_90_) of the mixtures (1:1, 1:2, and 2:1 respectively) were estimated as follows: 0.065–0.001 g·L^−1^ (1.651–0.011 g·L^−1^), 0.543–0.012 g·L^−1^ (6.553–0.028 g·L^−1^), and 0.387–0.015 g·L^−1^ (6.361–0.059 g·L^−1^) 48 h to 8 days after treatment.

The order of the tested BIs according to efficacy (LC) is summarized as follows: mixture (1:1), NeemAzal T/S (Trifolio-M GmbH, Lahnau, Germany), mixture (1:2), mixture (2:1), and Rock Effect (Agro CS a.s., Česká Skalice, Czech Republic). The improved efficacy of the mixtures indicates an obvious synergistic effect.

## 3. Discussion

This experiment involved testing the insecticidal activity of NeemAzal T/S (Trifolio-M GmbH, Lahnau, Germany) (neem oil), Rock Effect (Agro CS a.s., Česká Skalice, Czech Republic) (karanja oil), and three binary mixtures. In the course of the experiment, it was observed that the larvae treated with the oils also showed a reduction in food intake, which resulted in little leaf damage compared to leaves in the control variant. This effect was not a primary target of the testing; nevertheless, antifeedancy has previously been reported for karanja oil [36] as well as neem oil [37].

The efficacy of neem oil was very good even at low concentrations such as 0.1–2 g·L^−1^ in our bioassays. The efficacy of neem products may vary with respect to concentrations of azadirachtin [38], and thus some authors have stated lower insecticidal activity against CPB [39,40]. However, that was not the case in our study, because the commercial product NeemAzal T/S (Trifolio-M GmbH, Lahnau, Germany) used in the bioassay contains 1% of the purified active ingredient Azadirachtin A, which is a very potent insect growth inhibitor [41] (reviewed in [19,42]). In the case of karanja oil, higher doses were needed to achieve the same mortality of CPB larvae as with neem oil. According to the literature, karanja oil is generally effective at higher doses, from 10 g·L^−1^ upwards [43,44,45]. Deshmukh and Borle [46] also mentioned that karanja oil has some limits for use at the farmer’s level, because its aqueous suspension is not as effective as that of neem. The higher efficacy of neem oil over karanja oil was also reported by Biswas et al. [47] in the case of *Helicoverpa armigera*. 

Zehnder and Warthen [37] found that azadirachtin caused mortality, and they also observed an antifeedant effect in the case of CPB. Feuerhake and Schmutterer [48] tested neem seed extract formulations and were able to achieve 100% mortality of CPB. Other observed effects of neem oil on CPB are disruption of egg hatching and larvae molting [49]. The repellent effects of neem oil were even observed in laboratory as well as in field conditions [50]. Kaethner [51] observed the effect of fitness reduction in the treated beetles and a lower fecundity of females. Murray et al. [52] confirmed the oviposition effects of citrus limonoids, which are structurally and functionally similar to azadirachtin. The effect of karanja oil on CPB has not been studied to date. 

With respect to other insect pests, synergistic effects were reported for both neem and karanja oils. Synergism refers to when the combined effect of two factors is greater than the sum of individual factors. It can occur between various insecticides [14], insecticides and fungicides [53], insecticides and poor nutritional conditions [54], and so on. For example, a synergism was found for the combination of neem oil and *Beauveria bassiana* on *Spodoptera litura* Fabricius [55], *Tribolium castaneum* [56], and aphids [57]. The combination of both oils with some pyrethrins shows synergism as well [36,58]. Synergism was also observed in the combination of neem and karanja oil by Kumar et al. [34], where both oils were found to be highly effective individually and also in combination against mites and aphids. In our case, the most significant synergistic effect was found for the mixture of neem and karanja oil in a 1:1 ratio. In addition, this effect appeared chronically and not acutely, and thus it is possible to use lower doses of insecticides. A notable observation in this experiment was that the individual neem and karanja oil treatments were less effective compared to the mixtures. For example, the toxicity (LC_90_) five days after treatment with the 1:1 mixture was almost 3-fold higher than for neem oil alone, and up to 28-fold higher than for karanja. The other mixtures showed synergism as well, although the effect was significantly weaker. Packiam and Ignacimuthu [35] came to the same conclusions when testing various combinations of neem and karanja oils against larvae of *Spodoptera litura*. Thus, it is clear that the strength of the synergistic effect is ratio dependent, and this is the first research paper to describe such a phenomenon in the case of CPB.

Neem and karanja oil seem to provide the perfect solution to all the problems of contemporary crop protection. Nevertheless, the possible cons should be discussed as well. Because it possesses a variety of insecticidal properties, even against some beneficial insects in the juvenile stage [59], the use of neem oil carries a potential risk. Any decision to use neem oil should therefore be made carefully. The same situation occurs in the case of karanja oil, which is considered a broad-spectrum insecticide [36]. On the other hand, Koss et al. [60] and Radkova et al. [61] found that beneficial arthropods in potato fields were more abundant when botanical and selective pesticides were sprayed in contrast to when chemical pesticides were used. Neem oil is considered environmentally friendly because it is free of chloramine, phosphorous, and nitrogen atoms, which are commonly found in synthetic pesticides. Moreover, using neem oil in the field can prevent some other pests from ovipositing [62], thus providing improved crop protection, and due to the synergistic effect of the compounds, a reduced dose is used, significantly reducing toxicity to non-target organisms while also reducing the cost of applications. Generally, neem products can be recommended for many integrated pest management (IPM) programs [63]. The effect of an insecticide based on a combination of neem and karanja oils (PONNEEM^#^) has already been tested against the hymenopteran parasitoid wasp *Trichogramma chilonis*, which was not affected from applications with up to a 0.5% concentration [35]. The recommended dose for NeemAzal T/S (Trifolio-M GmbH, Lahnau, Germany) in potatoes is 2.5 L for 300–700 L of water/ha (0.8–0.35% concentration). Because the 1:1 mixture was almost 3 times more effective, we should theoretically recommend the use of a 0.3–0.1% concentration for field application, and thus parasitoids should not be affected. 

Field efficacy, however, will still need to be verified by a series of tests before its implementation, because some authors [64] have demonstrated the low stability of neem oil under field conditions due to photodegradation. This issue can be resolved theoretically via nano and micro encapsulation, which improves the stability and efficacy of oils exposed to UV light [65]; on the other hand, this solution is more suitable for soil applications of neem seed oil [66]. Moreover, the cost of such a product will be affected as well. The greater variability of karanja oil efficacy against CPB larvae reveals that this compound is less reliable than neem oil, especially when acute toxicity is concerned. However, Kumar and Singh [36] stated that the persistence of karanja oil is greater than for other botanical insecticides. In addition, the karanja extract is a highly effective UV absorbent [67] and is now being used in cosmetics as a component of modern sunscreen preparations, even for humans. As Wanyika et al. [68] have indicated, stabilization can be developed by adding solar radiation protectants, and therefore the combination of neem and karanja oils seems to be a perfect match, because what is missing in one is replaced by the other.

## 4. Materials and Methods 

### 4.1. Insecticides

Two botanical insecticides that are suitable for organic farming were used in the bioassays. The first one (NeemAzal T/S) (Trifolio-M GmbH, Lahnau, Germany) was tested at five different doses and the second one (Rock Effect) (Agro CS a.s., Česká Skalice, Czech Republic) was tested at six different doses. Both substances were tested as contact insecticides.

NeemAzal^®^ T/S (Trifolio-M GmbH, Lahnau, Germany) is a commercial formulation of the seed kernel extract of the tree *Azadirachta indica* (A.) Juss., which contains 1% of the purified active ingredient Azadirachtin A. It is generally used as a 0.3% to 0.5% aqueous solution. In the following text, the term “neem oil” is used.

Rock Effect (Agro CS a.s., Česká Skalice, Czech Republic) is a commercial formulation of *Pongamia pinnata* (L.) Pierre oil. The oil content is declared as 868.5 g·L^−1^. It is generally used as a 1% to 3% aqueous solution. In the following text, the term “karanja oil” is used.

### 4.2. Insects and Plant Material

Newly enclosed second instar larvae of *Leptinotarsa decemlineata* (Say) (Coleoptera: Chrysomelidae) were obtained from a colony reared on potato, *Solanum tuberosum*, cv. Agria, in a climate chamber at 22 ± 1 °C, a relative humidity of 40–60%, and a 16:8 (L:D) photoperiod. The colony was established from eggs and adults collected from a potato culture at a local field of the Crop Research Institute in Prague, Czech Republic (50°05′N, 14°18′E, 340 m a.s.l.) in June and August 2019.

### 4.3. Laboratory Bioassay

A potato leaf with five to seven leaflets was inserted into floral foam supporting moisture (Oasis, Belgium) and placed in a plastic box (16 × 11 × 7 cm). Twenty to twenty-five individuals (L2) of CPB were transferred carefully using a fine brush on each potato leaf. The tested BIs were dissolved in water right before use. NeemAzal T/S (Trifolio-M GmbH, Lahnau, Germany), Rock Effect (Agro CS a.s., Česká Skalice, Czech Republic), and their binary mixtures in three ratios (1:1, 2:1, and 1:2, by volume) were tested. For each BI, at least five concentrations that resulted in more than 0% and less than 100% mortality, based on preliminary assays, were used. The concentration series were 0.1–2 g·L^−1^ for NeemAzal T/S (Trifolio-M GmbH, Lahnau, Germany), 2–25 g·L^−1^ for Rock Effect (Agro CS a.s., Česká Skalice, Czech Republic), 0.05–2 g·L^−1^ for the 1:1 mixture, 0.1–15 g·L^−1^ for the 2:1 Rock Effect:Neem Azal (RE:NA) mixture, and 0.05–5 g·L^−1^ for the 1:2 (RE:NA) mixture. To obtain the LC value, the insecticidal activity of the concentration series of each insecticide was tested under laboratory conditions. 

Once the larvae began to feed on the leaves, the prepared solutions were sprayed on both sides of the leaves using a hand sprayer. The control variant was sprayed with water only. Each variant was performed in three repetitions. The experiment was run in a climate chamber set at 22 ± 1 °C and 69% ± 6% relative humidity. The mortality rate was evaluated at 48 h, 5 days, and 8 days later. Individuals unable to right themselves when disturbed were counted as dead.

### 4.4. Statistical Analysis

Abbott’s formula was used to correct the data for control mortality [69]. Probit analysis [70] was used to estimate the doses needed to cause 50% mortality (LC_50_) and 90% mortality (LC_90_), as well as the associated 95% confidence limits for each BI, including binary mixtures, using BioStat (version 5.9.8.5). 

## 5. Conclusions

Because of their natural origin and environmental friendliness, botanical pesticides currently have great potential. Furthermore, botanical pesticides are also very potent insecticides and, due to their composition, they can help to fight the global problem of insects developing resistance to insecticides.

Both of the tested BIs (insecticides based on neem oil and karanja oil) were efficient against *L. decemlineata* larvae at different concentrations. However, the efficacy of karanja oil against CPB larvae showed higher variability than for neem oil, on account of the high azadirachtin content of the commercial neem product and also the slightly different mode of action of both oils. Our experiment demonstrated a synergistic effect of neem and karanja oils against CPB larvae under laboratory conditions—one that was ratio-dependent. The most potent mixture (1:1 ratio) was equally, or even more effective, than neem oil itself. This effect intensified with respect to exposure time and appeared chronically. Five days after application of the mixture, LC_90_ values were 3-fold higher for neem oil and up to 28-fold higher for karanja oil. Due to these results, the recommended field dose of the mixture was estimated as high as 1.4 L in 500 L of water/ha (0.3%). Nevertheless, further verification in field conditions of the results achieved by this experiment is necessary in order to reach reliable conclusions and implement them into practice, and the effect on beneficial and non-target organisms should be verified.

## Figures and Tables

**Table 1:** Toxicity of botanical insecticides (Bis) and their mixtures against *Leptinotarsa decemlineata* larvae. LC_50_ and LC_90_: concentration causing 50% and 90% mortality of insects, respectively. CI_95_: 95% confidence intervals, insecticide activity is considered significantly different when the 95% CI fails to overlap. Chi = Chi-square value, significant at *p* < 0.05 level.

Insecticides	Days after Treatment	LC^50^	CI^95^	LC^90^	CI^95^	Chi-Square	*p*-Value
Neem Azal T/S	2	0.075 ± 0.011	0.066–0.101	0.618 ± 0.215	0.372–1.323	0.988	0.804
	5	0.021 ± 0.002	0.011–0.021	0.105 ± 0.011	0.082–0.156	2.031	0.565
	8	0.005 ± 0.001	0.003–0.008	0.029 ± 0.004	0.022–0.041	0.007	0.999
Rock Effect	2	0.582 ± 0.093	0.344–0.858	1.692 ± 0.428	1.112–4.319	2.316	0.677
	5	0.371 ± 0.071	0.190–0.553	1.032 ± 0.263	0.672–2.813	5.065	0.281
	8	0.259 ± 0.054	0.119–0.379	0.774 ± 0.176	0.523–1.165	1.821	0.768
Rock Effect and NeemAzal T/S 1:2	2	0.543 ± 0.151	0.367–0.611	6.553 ± 2.441	5.369–7.921	6.591	0.252
	5	0.021 ± 0.005	0.015–0.031	0.151 ± 0.027	0.094–0.211	5.398	0.369
	8	0.012 ± 0.001	0.012–0.017	0.028 ± 0.002	0.024–0.034	0.677	0.954
Rock Effect and NeemAzal T/S 1:1	2	0.065 ± 0.016	0.042–0.113	1.651 ± 0.325	1.354–1.923	2.088	0.552
	5	0.008 ± 0.015	0.001–0.060	0.037 ± 0.023	0.016–0.065	1.507	0.681
	8	0.001 ± 0.001	0.002–0.026	0.011 ± 0.001	0.007–0.016	0.052	0.996
Rock Effect and NeemAzal T/S 2:1	2	0.387 ± 0.052	0.302–0.512	6.361 ± 2.585	3.391–7.182	5.126	0.401
	5	0.053 ± 0.002	0.012–0.091	0.267 ± 0.085	0.168–0.378	3.771	0.582
	8	0.015 ± 0.002	0.011–0.019	0.059 ± 0.007	0.047–0.077	0.336	0.996
